# Successful Prolonged Mechanical CPR in a Severely Poisoned Hypothermic Patient: A Case Report

**DOI:** 10.1155/2012/381798

**Published:** 2012-08-23

**Authors:** Alberto Piacentini, Maurizio Volonte', Marcello Rigamonti, Elisa Guastella, Mario Landriscina

**Affiliations:** ^1^Anesthesiology-118 Service AAT Como, Italy; ^2^Azienda Ospedaliera Sant'Anna, via Ravona, 1 22020 San Fermo della Battaglia (CO), Italy; ^3^AAT 118 Como, 22079 Villa Guardia, Como (CO), Italy; ^4^Emergency Nurse AAT 118 Como, 22079 Villa Guardia, Como (CO), Italy; ^5^Intensive Care-Hems Service, 118 Como, 22079 Villa Guardia, Como (CO), Italy

## Abstract

Mechanical cardiopulmonary resuscitation (m-CPR) devices are an alternative to manual CPR, but their efficacy has been subject to debate. We present a case of a patient with full-neurologic recovery after prolonged m-CPR. The patient presented with severe hypothermia (internal temperature 24°C) and poisoning (sedatives/hypnotics). Hepatic perfusion and metabolism are considered keys to restore spontaneous circulation. During this period no problems related to the device or patient positioning were encountered. Delivery of high-quality CPR and prolonged resuscitation were achieved. We confirm that ventilations asynchronous with chest compressions can be a problem. Reduction in chest measurements can hamper lung ventilation. A synchronous mode of manual ventilation (30 : 2) seems to be the best solution. The patient had an initial period of manual CPR. No damage to any organ or structure was noted. This case is of further interest because our EMS helicopters can fly 24 hours a day and m-CPR devices could play an important role as a “bridge” in patients when active rewarming by cardiopulmonary bypass is indicated (CPB).

## 1. Introduction

We report the case of a patient with full-neurologic recovery following prolonged mechanical cardiopulmonary resuscitation (m-CPR) with an AutoPulse.

Mechanical resuscitation devices have been used by many emergency medical services (EMS) throughout Europe. M-CPR devices are a valid alternative to manual chest compressions, showing an increase in quality of CPR performed by two-member response teams, and during diagnostic or therapeutic procedures such as CT scan or PCI. They may also be used for ongoing CPR during patient transportation, especially if a potentially reversible cause is suspected [1, 2]. M-CPR has been associated with an increased risk of internal injuries when compared to manual resuscitation. It is not clear whether this is due to m-CPR itself or the combination of m-CPR with previous manual CPR. 

In 2006 the Czech Republic EMS system compared the Lucas and the AutoPulse m-CPR devices and found the incidence of CPR-associated injuries to be similar [3].

Two recent cases of m-CPR used for cardiac arrest secondary to massive pulmonary embolism have suggested caution due to increased risk of liver injury and bleeding. The mechanism is thought to be an augmented portal vein blood pressure. These two patients received also manual CPR [4]. 

## 2. Case

At 9:01 pm, a call was received in our 118 Center (911 Center) from a woman who reported her 42-year-old sister was no longer answering the phone and that a suicide attempt was suspected. The lights in the patient's apartment were turned on, and she had been last heard from at approximately 5:00 pm that afternoon.

At 09:23 pm, the responding BLS team reported to our Regional Emergency Dispatch Center (Articolazione Aziendale Territoriale: A.A.T.118-Como, northern Italy) that they had made first patient contact following a forced entry by firefighters. 

Scene size up: patient unresponsive, airway patent, breathing shallow and slow, carotid pulse present but weak, radial pulse absent, systolic blood pressure 60 mm Hg, and heart rate 35 beats per minute. The patient was lying on a marble floor in a right lateral decubitus position with extremely cold skin.

Our EMS teams do not have the capability of measuring extremes of body temperature, but the patient appeared hypothermic and had signs of stage one decubitus ulcers (surface reddening of the skin) of the right upper arm and right waist.

**Figure 1:**
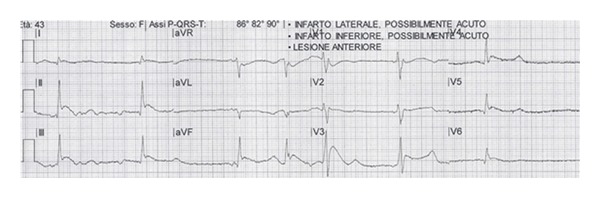
12 lead EKG at EMS arrival (typical Osborn waves, also known as camel-hump sign, late delta wave, or hypothermic wave. Deflections are more commonly observed in leads II, III, AVF, V5, and V6).

She was placed on a monitor and gently moved in a supine neutral position taking all precautions suggested in cases of deep hypothermia. A 12 lead EKG showed typical Osborn waves: (Figure 1).

09:50 pm: despite the efforts taken, the patient developed ventricular fibrillation (VF) and was immediately converted to a slow sinus rhythm by an immediate countershock of 200 Joules.

At this time an ALS team secured the patient's airway via endotracheal intubation. GCS 3: cardiac frequency 50 beats per minute. The patient developed recurrent VF, manual CPR was started, and transport was initiated.

At the same time another advanced life support team (fast medical car) was dispatched with a mechanical chest compression device to the emergency department.

10:14 pm: patient arrived in a “red code” priority to hospital. An AutoPulse was immediately positioned while manual CPR was continued, and then mechanical compressions were initiated. 

10:20 pm: an arterial catheter was easily positioned during m-CPR and serial arterial blood gas samples were collected (Table 1).

The esophageal temperature probe was inserted showing an initial core temperature of 24°C.

After a gastric tube was positioned, a large amount of gastric content was retrieved for toxicology tests.

A central venous catheter and urinary catheter were also inserted.

Due to the unavailability of active internal warming by cardiopulmonary bypass (CPB) in our institution (“*Ospedale S. Anna*”, Como Italy) and a long interfacility transfer to a CPB referral center (“*Ospedali Riuniti*”, Bergamo Italy), a direct transfer did not seem safe (distance more than 49 miles). It was decided to sustain cardiac circulation and to begin both active internal and external rewarming. An attending physician was dedicated to continuously monitor the AutoPulse and synchronization of ventilation.

 Body temperature slowly increased to 27.8°C and the presence of a spontaneously beating heart was recorded at 11:40 pm. Total m-CPR time was eighty minutes. In this period no problems related to battery power, compressing belt, or need for patient repositioning on the device were encountered.

The patient was transferred to the ICU. No abnormalities were found by chest radiography or sonography.

**Figure 2:**
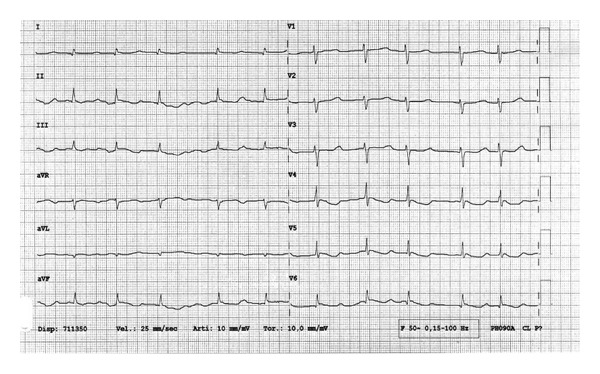
12 leads EKG after prolonged m-CPR.

The CPK-MB plasma peak was 1 ng/mL. A 12 lead EKG showed only minimal ischemic changes: diffuse *T* wave inversion (Figure 2).

In the postresuscitative period we did not find any pulmonary lesion (the highest inspired oxygen fraction was 0.35). The patient required mechanical ventilatory support only for the time necessary to clear the ingested substances. She did not develop any ventilator acquired pneumonia (VAP). She did not show any transient or persistent neurological deficit upon awakening. Cerebral CT scan was negative for ischemic brain damage.

## 3. Conclusion

This case demonstrates that the immediate availability of m-CPR is an extremely powerful resource that provides clinically effective compressions while conserving manpower and associated logistics. 

## 4. Indications

In our patient, three strong advanced life support resuscitation challenges were met simultaneously: delivery of high quality CPR, need for prolonged resuscitation, and association of poisoning and severe hypothermia.

Acute poisoning (any substance) is a specific condition that cannot be relieved by CPR alone. In this sense m-CPR served as a good temporary “bridge” until the return of spontaneous circulation. 2010 ALS guidelines outline this particular situation, especially in young patients, where hepatic perfusion and metabolism are the fundamental keys. Severely hypothermic patients can present with a rigid chest wall which hinders manual CPR. Load distributing band (LDB) CPR devices, which deliver a uniform pressure on a large chest wall surface, can be a possible solution.

## 5. Possible Biases

In this particular case, severe hypothermia associated with substance abuse (sedative/hypnotics) could have provided a form of cerebral cortical protection for this patient, when compared to a normothermic poisoned patient. 

## 6. Benefits Observed

The ALS team leader was able to focus on the overall care of the patient, while delivering good quality CPR (as intended in ALS guidelines 2010), checking continuously for proper patient positioning of the device [1]. 

## 7. Lessons Learned and Suggestions

- As previously demonstrated by other authors during m-CPR, asynchronous ventilation can be a problem. The reduction in anterior-posterior chest measurement hampers lung ventilation. Reverting to a synchronous manual ventilation modality (30 : 2) is beneficial [5].
- Checking frequently for any mismatch between thoracic surface anatomy and load distributing band position ensured good quality CPR without interruption in resuscitation and prevented any injury. Our patient did not receive any thrombolytic therapy but had an initial brief period of manual CPR. She did not show any damage to any internal structure (i.e., rib cage, lungs, pleura, mediastinum, or abdominal organs) [6].
- The m-CPR device we employed relies on a load distributing band (LDB) which can be used in a hypothermic low-compliance chest wall without further Injury.
- In our region HEMS can provide interfacility flights under instrument flight rules (IFR) 24 hours a day.

From the perspective of a “*Hub & Spoke*” system, our m-CPR devices are currently undergoing aviation certification and could play, together with helicopters, a pivotal role as temporary “bridging devices” during transfer of patients who are candidates for cardiopulmonary bypass (CPB) directly from the scene or from nearby local hospitals [7]. Studies are needed to better understand which subcategories of this class of patients would benefit from such a strategy.

## Figures and Tables

**Table 1:** ABG values.

ABG value	22 : 29 (t0)	22 : 59 (t0 + 30^′^)	23 : 41 (t0 + 72^′^)	00 : 56 (t0 + 147^′^)	02 : 57 (t0+268′)	09 : 10 (t0+641′)
Temp. ^°^C	24.0	24.0	28.0	31.0	33.5	37.0
pH	7.031	6.96	7.056	7.103	7.33	7.335
pO_2_	37.5	88	39.5	81.0	91.1	193
pCO_2_	50.1	42.8	29.7	22.9	28.5	24.4
K^+^	2.3	4.2	3.9	3.3	3.2	3.8
HCO_3_^−^	10.6	8.8	8.6	8.9	12.6	15.5
Lac	8.8	8.7	9.5	11.7	13.3	13.2
